# *Escherichia coli* O157 Infection and Secondary Spread, Scotland, 1999–2008

**DOI:** 10.3201/eid1703.100167

**Published:** 2011-03

**Authors:** Mary E. Locking, Kevin G.J. Pollock, Lesley J. Allison, Linda Rae, Mary F. Hanson, John M. Cowden

**Affiliations:** Author affiliations: Health Protection Scotland, Glasgow, Scotland (M.E. Locking, K.G.J. Pollock, L. Rae, J.M. Cowden);; Scottish *E. coli* O157/VTEC Reference Laboratory, Edinburgh, Scotland (L.J. Allison, M.F. Hanson)

**Keywords:** Bacteria, Escherichia coli O157, secondary spread, outbreak, hemolytic uremic syndrome, bloody diarrhea, asymptomatic, sorbitol-fermenting, Scotland, dispatch

## Abstract

To determine the proportion of *Escherichia coli* O157 cases in Scotland attributable to secondary spread, we analyzed data obtained through entire-population enhanced surveillance. We identified 11% of cases as secondary. Secondary cases in single households were younger than secondary cases in outbreaks affecting >1 household and had similar risk for hemolytic uremic syndrome.

*Escherichia coli* O157 remains a substantial public health challenge worldwide, particularly because of its association with hemolytic uremic syndrome (HUS) (*1**,**2*). The low infectious dose (*3*) exacerbates its potential to cause secondary spread and large outbreaks (*4**–**10*). Most published information about secondary spread derives from outbreaks (*7**,**9**–**12*) or from subgroups or settings not necessarily generalizable to whole populations (*4**,**11*). Rates of secondary cases range from 4% to 16% (*4**,**9*).

Associations with increased transmission include presence of siblings, young age of persons with primary or potential secondary cases (*4**,**10**,**11*), and waterborne compared with foodborne transmission in outbreaks (*12*). Scotland consistently reports higher rates of *E. coli* O157 infection than many other countries (Figure 1); *E. coli* O157 was identified in 81% of HUS cases (*13*). Although large outbreaks have occurred (*7**,**8*), most infections in Scotland are apparently sporadic (*14*) (Figure 2).

**Figure 1:**
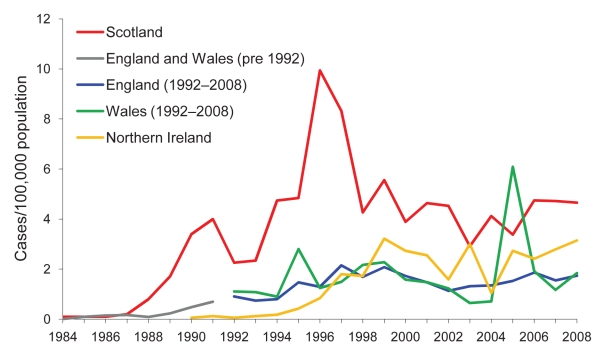
Rates per 100,000 population of laboratory-confirmed culture-positive *Escherichia coli* O157 cases, by country, United Kingdom, 1984–2008. Data outside Scotland courtesy of Health Protection Agency London, and Public Health Agency Belfast; figures for England, Wales, and Northern Ireland are verotoxin-positive cases only. Data for 2008 outside Scotland are provisional.

**Figure 2:**
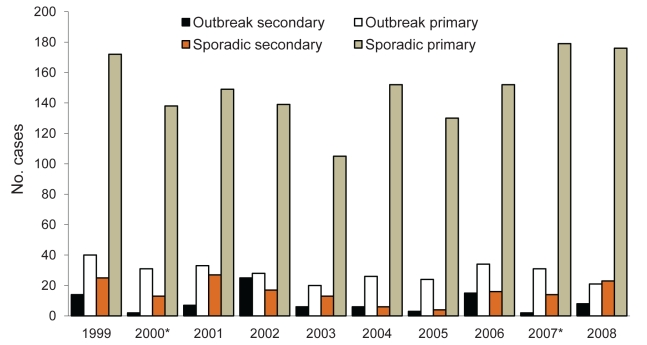
Secondary symptomatic or primary symptomatic laboratory-confirmed *Escherichia coli* O157 cases, by outbreak or sporadic occurence, Scotland, 1984–2008.

## The Study

In 1999, Health Protection Scotland (HPS), in close collaboration with the Scottish *E. coli* O157/VTEC Reference Laboratory (Edinburgh, Scotland), established enhanced surveillance of *E. coli* O157 covering the entire population. HPS defines a case as a single-person infection episode with laboratory confirmation of infection as either culture positive or serum positive for *E. coli* O157. The term case refers to symptomatic and asymptomatic persons, i.e., patients and nonpatients.

HPS compiles standardized datasets for all cases, integrating microbiologic, epidemiologic, demographic, and exposure data; datasets include direct clinical reports of HUS under enhanced surveillance of thrombotic microangiopathies since 2003 (*13*) and detailed symptom descriptions since 2004. Secondary cases are defined as those symptomatic cases from whose onset date and an assumed incubation period (<14 days) we can infer that contact with a confirmed case was more likely than any other exposure to be the source of infection, and whose onset was >2 days after onset in the contact case. Cases having onset within 2 days after onset in a contact case are defined as co-primaries.

HPS surveillance systems collect information about general outbreaks, i.e., those affecting members of >1 household or residents of an institution. Other cases are therefore either sporadic or occur among members of a single household. For ease of comprehension, we refer to cases in general outbreaks as outbreak cases and cases or clusters restricted to a single household as sporadic cases, irrespective of whether they are secondary or primary cases. We analyzed data for all cases reported to HPS during 1999–2008 using χ^2^ and Mann-Whitney tests and considered p<0.05 as significant.

From January 1, 1999, through December 31, 2008, a total of 2,228 *E. coli* O157 cases were reported to HPS (mean 223 annually); the mean annual incidence rate was 4.4 cases per 100,000 population (Table 1; Figure 1). Ages of the 2,228 cases ranged from 4 months to 97 years (median 21 years). A minority of all cases (202/2,228 [9%]) were asymptomatic, in similar proportions annually (p = 0.44) (Table 1). All 1,118 cases reported 2004–2008 provided symptom details; 660 (59%) had bloody diarrhea. Varying proportions of cases each year were hospitalized (mean 41%; p<0.005) or had illness progressing to HUS (196/2,228; mean 9%; p = 0.03) (Table 1). HUS occurred more often in cases reporting bloody diarrhea than nonbloody diarrhea (14% vs. 3%, p<0.0005), suggesting that bloody diarrhea may be a better predictor of progression to HUS than was previously apparent in Scotland.

**Table 1:** Selected characteristics and health outcomes of laboratory-confirmed *Escherichia coli* O157 cases, Scotland, 1999–2008*

Characteristic	No. (%) cases, n = 2,228†	Range per year, % (p value)
Average reports per year, all cases	223	153–282
Average annual incidence per 100,000 population	4.4	3.0–5.6
Symptomatic cases	2,026 (91)	89–94 (0.44)
Asymptomatic cases	202 (9)	6–11 (0.44)
Secondary case‡	246 (12)‡	4–20 (<0.0005)
Primary case‡	1,780 (888)‡	80–96 (<0.0005)
Bloody diarrhea§	660 (59)§	56–62 (0.80)
Hospitalized case-patients	902 (41)	29–48 (<0.0005)
Hemolytic uremic syndrome	196 (9)	6–14 (0.03)
Outbreak case	441 (20)	13–27 (<0.0005)
Sporadic case	1,787 (80)	73–87 (<0.005)
Sporadic and symptomatic¶	1,650 (92) ¶	90–95 (0.84)
Sporadic, symptomatic, and secondary#	158 (10)#	3–15 (<0.005)
Sporadic, symptomatic, and primary#	1,492 (90)#	85–97 (<0.005)
*Cases include symptomatic and asymptomatic persons. †Unless otherwise indicated. ‡Denominator = 2,026 symptomatic cases 1999–2008. §Denominator = 1,118 cases reported 2004–2008, all with symptom details available (1,022 symptomatic and 96 asymptomatic cases). ¶Denominator = 1,787 sporadic cases. #Denominator = 1,650 symptomatic sporadic cases.		

Secondary cases constituted 246/2,026 (12%) of the symptomatic cases (11% of all cases), with proportions varying annually (range 4%–20%; p<0.0005) (Table 1; Figure 2), apparently independent of incidence rates. Secondary cases were younger than primary cases (median 13 years vs. 20 years; p<0.0005). Fewer secondary than primary cases had bloody diarrhea (54% vs. 66%; p = 0.02) (Table 2), but secondary cases with bloody diarrhea were younger than primary cases with bloody diarrhea (median 13 years vs. 26 years; p<0.03), perhaps reflecting lower thresholds for screening younger contacts. Secondary cases accounted for 12% of all HUS cases, and their likelihood of having HUS was similar to that of primary cases (p = 0.95) (Table 2). Mean time between onset in primary and secondary cases was 8 days (range 3–24 days); the longer times occurred when primary cases were symptomatic for >14 days. Child-to-child transmission accounted for 72% of secondary cases, child-to-adult for 19%, and adult-to-adult for 9%.

**Table 2:** Selected characteristics and health outcomes of secondary or primary symptomatic laboratory-confirmed *Escherichia coli* O157 cases, Scotland, 1999–2008

Characteristic	No. (%) cases, n = 2,026		p value
	Secondary cases, n = 246*	Primary cases, n = 1,780*	
Case age <10 y	116 (47)	623 (35)	<0.0005
Female sex	147 (60)	958 (54)	0.07
Bloody diarrhea†	52 (54)†	608 (66)†	0.02
Hospitalized	82 (33)	816 (46)	<0.0005
Illness progressed to hemolytic uremic syndrome	24 (10)	172 (10)	0.95
Sporadic case	158 (64)	1492 (84)	<0.0005
Outbreak case	88 (36)	288 (16)	<0.0005
Outbreak case, with bloody diarrhea‡	25 (28)‡	81 (28)‡	0.93
Outbreak case, hospitalized‡	32 (36)‡	128 (44)‡	0.22

*Unless otherwise indicated. †Denominator = 1,022 symptomatic cases reported 2004–2008, all with symptom details available (97 secondary and 925 primary cases). ‡Denominator = 376 outbreak symptomatic cases (88 secondary and 288 primary cases).

Most cases (1,787/2,228 [80%]) were sporadic (Table 1; Figure 2). Similar proportions of sporadic and outbreak cases (p = 0.89) had illness progressing to HUS. Of the 1,650 sporadic cases who were symptomatic, 158 (10%) were secondary cases (Table 2). Sporadic and outbreak secondary cases had the same risk for HUS (p = 0.97), but sporadic secondary cases were younger (median 9 years vs. 26 years; p<0.04), highlighting the need to prevent transmission within single households.

Outbreak cases constituted 441/2,228 (20%) cases, but proportions varied annually (range 13%–27%; p<0.0005) (Table 1; Figure 2). Of the 88 secondary cases in outbreaks, 57 (65%) lived in the same household as the associated primary case; the remainder were contacts either in institutions or in linked second households. The 441 outbreak cases comprised 104 separate outbreaks. Secondary cases were identified in 40 (42%) of the 95 outbreaks that occurred in Scotland, with an average ratio of secondary to primary cases of 1.3:1.

## Conclusions

The reasons for high incidence rates of *E. coli* O157 in Scotland are undoubtedly complex and multifactorial. Influences affecting real incidence may include the relative population densities of livestock and humans and reliance on private water supplies (*8*).

Ascertainment of secondary cases in Scotland, which appeared to have a greater role in our study than may have been commonly assumed previously, may however be particularly affected by artifactual influences, such as more assiduous contact tracing resulting from heightened awareness, perhaps triggered by a combination of large outbreaks, a national task force, and enhanced surveillance (*7**,**8*). This possibility necessitates caution in extrapolating our findings on secondary case incidence to other countries. Such tracing and confirmation of infection is valuable in controlling household transmission as well as outbreaks, and for clinical management (*10**,**11*). Alternatively, some secondary cases will undoubtedly be missed, or misclassified as primary cases.

Because most secondary cases in Scotland are apparently sporadic, our findings also reinforce the need for low thresholds for suspecting infectious etiology in acute diarrhea (particularly if bloody), irrespective of (and without waiting to discover) whether cases are part of outbreaks (*2**,**11*). Patients need immediate advice about infection control in the home, accompanied by immediate stool sampling and monitoring (e.g., blood parameters), not just for primary or index cases but also for their contacts (*9**–**11*). We must continually raise professional and public awareness of secondary spread and measures needed to reduce it, and to ameliorate health outcomes (*2*,*9**,**10*). We should also maintain preventive strategies targeting the livestock-related risks strongly associated with sporadic infection (*8**,**14**,**15*).

We believe enhanced surveillance in Scotland provides uniquely valuable information, particularly about secondary transmission, because data derive from the entire population and are standardized and long term. They remove reliance on extrapolating from studies of outbreaks, subgroups, or other countries, which may use substantially different methods and settings. Our data also permit robust long-term analysis, which is central to identifying whether differences in incidence or epidemiology are real or artifactual, and whether those differences are meaningful for public health.

We strongly recommend increased efforts to prevent secondary transmission within individual households. This would reduce not only the overall health and social costs of *E. coli* O157 infection but also the number of, and distress to, HUS cases attributable to secondary spread.
